# Genome sequence of the mud-dwelling archaeon *Methanoplanus limicola* type strain (DSM 2279^T^), reclassification of *Methanoplanus petrolearius* as *Methanolacinia petrolearia* and emended descriptions of the genera *Methanoplanus* and *Methanolacinia*

**DOI:** 10.4056/sigs.5138968

**Published:** 2014-03-15

**Authors:** Markus Göker, Megan Lu, Anne Fiebig, Matt Nolan, Alla Lapidus, Hope Tice, Tijana Glavina Del Rio, Jan-Fang Cheng, Cliff Han, Roxanne Tapia, Lynne A. Goodwin, Sam Pitluck, Konstantinos Liolios, Konstantinos Mavromatis, Ioanna Pagani, Natalia Ivanova, Natalia Mikhailova, Amrita Pati, Amy Chen, Krishna Palaniappan, Miriam Land, Shanmugam Mayilraj, Manfred Rohde, John C. Detter, Boyke Bunk, Stefan Spring, Reinhard Wirth, Tanja Woyke, James Bristow, Jonathan A. Eisen, Victor Markowitz, Philip Hugenholtz, Nikos C. Kyrpides, Hans-Peter Klenk

**Affiliations:** 1 Leibniz Institute DSMZ - German Collection of Microorganisms and Cell Cultures, Braunschweig, Germany; 2 DOE Joint Genome Institute, Walnut Creek, California, USA; 3 Los Alamos National Laboratory, Bioscience Division, Los Alamos, New Mexico, USA; 4 T. Dobzhansky Center for Genome Bionformatics, St. Petersburg State University, St. Petersburg, Russia; 5 Algorithmic Biology Lab, St. Petersburg Academic University, St. Petersburg, Russia; 6 Biological Data Management and Technology Center, Lawrence Berkeley National Laboratory, Berkeley, California, USA; 7 Oak Ridge National Laboratory, Oak Ridge, Tennessee, USA; 8 MTCC – Microbial Type Culture Collection & Gene Bank, CSIR-Institute of Microbial Technology, Chandigarh, India; 9 HZI – Helmholtz Centre for Infection Research, Braunschweig, Germany; 10 University of Regensburg, Microbiology – Archaeenzentrum, Regensburg, Germany; 11 University of California Davis Genome Center, Davis, California, USA; 12 Australian Centre for Ecogenomics, School of Chemistry and Molecular Biosciences, The University of Queensland, Brisbane, Australia; 13 Department of Biological Sciences, King Abdulaziz University, Jeddah, Saudi Arabia

**Keywords:** anaerobic, motile, mesophilic, methanogen, swamp, improved-high-quality draft, *Methanomicrobiaceae*, GEBA

## Abstract

*Methanoplanus limicola* Wildgruber *et al*. 1984 is a mesophilic methanogen that was isolated from a swamp composed of drilling waste near Naples, Italy, shortly after the *Archaea* were recognized as a separate domain of life. *Methanoplanus* is the type genus in the family *Methanoplanaceae*, a taxon that felt into disuse since modern 16S rRNA gene sequences-based taxonomy was established. *Methanoplanus* is now placed within the *Methanomicrobiaceae*, a family that is so far poorly characterized at the genome level. The only other type strain of the genus with a sequenced genome, *Methanoplanus petrolearius* SEBR 4847^T^, turned out to be misclassified and required reclassification to *Methanolacinia*. Both, *Methanoplanus* and *Methanolacinia,* needed taxonomic emendations due to a significant deviation of the G+C content of their genomes from previously published (pre-genome-sequence era) values. Until now genome sequences were published for only four of the 33 species with validly published names in the *Methanomicrobiaceae*. Here we describe the features of *M. limicola*, together with the improved-high-quality draft genome sequence and annotation of the type strain, M3^T^. The 3,200,946 bp long chromosome (permanent draft sequence) with its 3,064 protein-coding and 65 RNA genes is a part of the ***G****enomic* ***E****ncyclopedia of* ***B****acteria and* ***Archaea***** project.

## Introduction

Strain M3^T^ (= DSM 2279 = ATCC 35062 = OCM 101) is the type strain of the species *Methanoplanus limicola* [1,2], one out of currently three species in the genus *Methanoplanus* [1,2]. Strain M3^T^ was originally isolated from the mud of a drilling swamp near Baia, Naples Area, Italy [1]. The genus name was derived from the Neo-Latin therm “*methanum*”, pertaining to methane, and the Latin adjective “*planus*”, meaning a flat plate, which refers to its flat cell morphology [1]. The species epithet was derived from the Latin word *limicola*, a dweller in the mud, inhabitant of a swamp [1]. When Wildgruber *et al*. described the type strain of the novel species in 1982 [1] they not only realized the striking similarity to the square-shaped flat bacterium that was reported two years earlier by Walsby [3], but also classified it as the type strain of the type species in the type genus of *Methanomicrobiales* Family III, ‘*Methanoplanaceae*’ [1]. However, when years later 16S rRNA sequences became available for phylogenetic analyses it became clear that the strains which represent the species *Methanoplanus* are closely related to *Methanomicrobiaceae* (including the genera *Methanomicrobium*, *Methanogenium*, and *Methanoculleus*). Since that time, the genus *Methanoplanus* is generally placed within the *Methanomicrobiaceae*, and *Methanoplanaceae* Wildgruber *et al*. 1984 has fallen into disuse [4], although the genus *Methanoplanus* was never formally reclassified. In the 31 years since strain M3^T^ was first characterized, only two follow-up projects have reported the use of *M. limicola* in comparative analyses; Ivanov and Stabnikova [5] used *M. limicola* for a study on the molecular phylogeny of methanogenic archaea based on the G+C content, and Liu *et al*. used the species in a study on air tolerance and water stress [6].

Here we present a summary classification and a set of features for *M. limicola* M3^T^, together with the description of the genomic sequencing and annotation.

## Classification and features

The single genomic 16S rRNA sequence of *M. limicola* M3^T^ was compared with the Greengenes database for determining the weighted relative frequencies of taxa and (truncated) keywords as previously described [7]. The most frequently occurring genera were *Methanoculleus* (51.9%), *Methanoplanus* (18.5%), *Methanogenium* (16.8%), *Methanosphaerula* (5.3%) and *Methanomicrobium* (3.7%) (52 hits in total). Regarding the two hits to sequences from members of the species, the average identity within HSPs was 99.9%, whereas the average coverage by HSPs was 92.8%. Regarding the five hits to sequences from other members of the genus, the average identity within HSPs was 96.6%, whereas the average coverage by HSPs was 95.0%. Among all other species, the one yielding the highest score was *M. endosymbiosus* (FR733674), which corresponded to an identity of 99.5% and an HSP coverage of 99.7%. (Note that the Greengenes database uses the INSDC (= EMBL/NCBI/DDBJ) annotation, which is not an authoritative source for nomenclature or classification.) The highest-scoring environmental sequence was EU420694 ('Archaeal and Kao-Mei Wetland clone KM07-Da-3'), which showed an identity of 95.7% and an HSP coverage of 98.0%. The most frequently occurring keywords within the labels of all environmental samples which yielded hits were 'temperatur' (4.7%), 'bioreactor' (4.4%), 'anaerob' (4.0%), 'methanogen' (3.3%) and 'archaeal' (2.9%) (198 hits in total) fit to the features known from the habitat of strain M3^T^. Environmental samples which yielded hits of a higher score than the highest scoring species were not found.

**Figure 1:**
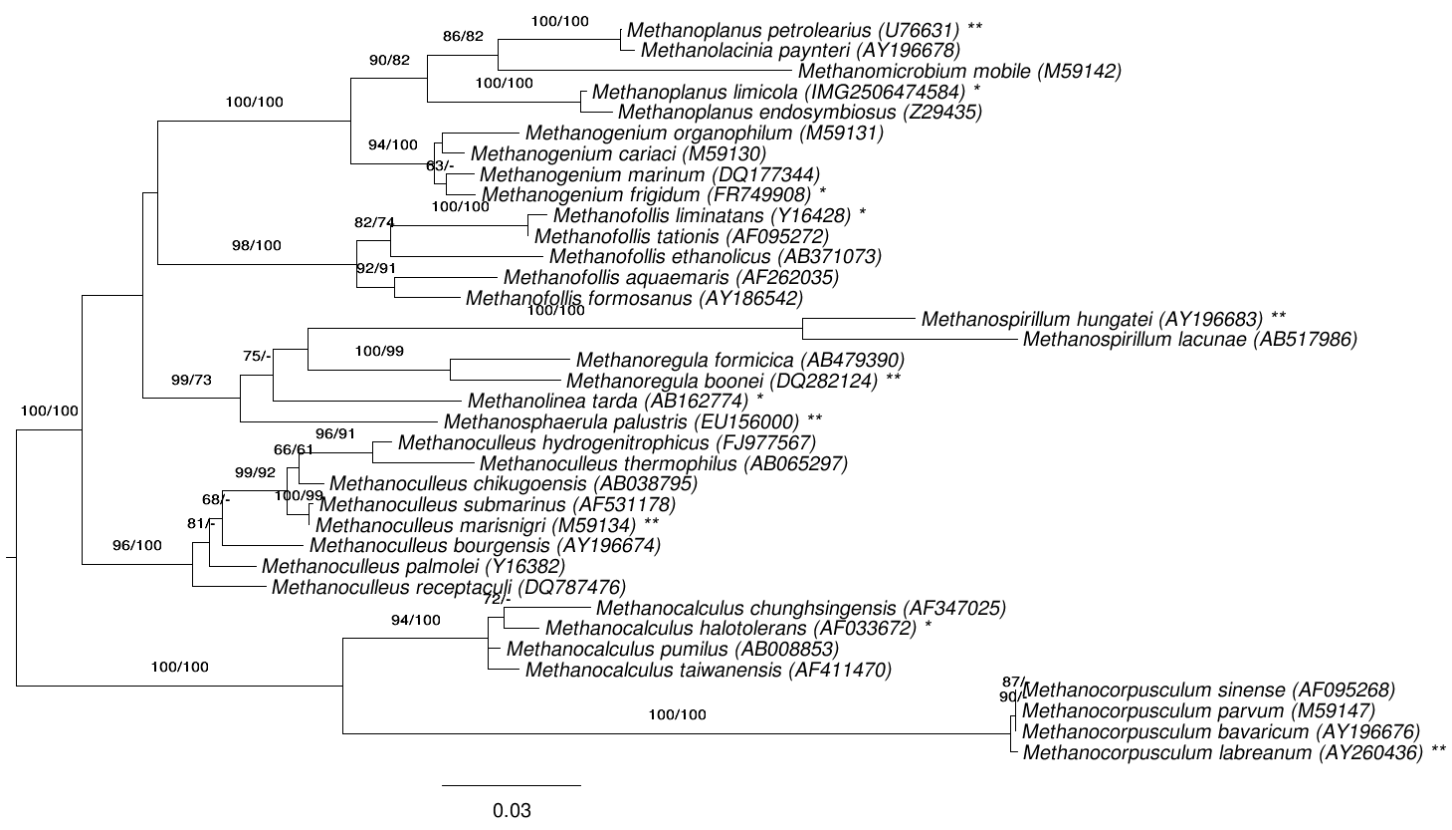
Phylogenetic tree highlighting the position of *M. limicola* relative to the type strains of the other species within the family *Methanomicrobiaceae*. The tree was inferred from 1,271 aligned characters of the 16S rRNA gene sequence under the maximum likelihood (ML) criterion and rooted as previously described [7]. The branches are scaled in terms of the expected number of substitutions per site. Numbers adjacent to the branches are support values from 250 ML bootstrap replicates [8] (left) and from 1,000 maximum-parsimony bootstrap replicates [9] (right) if larger than 60%. Lineages with type-strain genome sequencing projects registered in GOLD [10] are labeled with one asterisk, those also listed as 'Complete and Published' with two asterisks [11-14] (for *Methanoregula boonei* and *Methanosphaerula palustris* see CP000780 and CP001338, respectively).

Figure 1 shows the phylogenetic neighborhood of *M. limicola* in a 16S rRNA based tree. The sequence of the single 16S rRNA gene copy in the genome does not differ from the previously published 16S rRNA sequence (M59143), which contains 23 ambiguous base calls.

The tree depicted in Figure 1 reveals discrepancies between the current classification of the group and 16S rRNA phylogenetic analysis, as the genus *Methanoplanus* appeared polyphyletic, with *M. petrolearius* appearing as sister group of *Methanolacinia payntneri* with maximum support. We conducted a constraint analysis as previously described [15], enforcing the monophyly of all genera (which only affects *Methanoplanus* in this dataset, see Figure 1). The best-known ML tree had a log likelihood of -7,097.90, whereas the best tree found under the constraint had a log likelihood of -7,144.12. The constrained tree was significantly worse than the globally best one in the Shimodaira-Hasegawa test as implemented in RAxML [8] (α = 0.01). The best-known MP trees had a score of 1,090, whereas the best constrained trees found had a score of 1,115 and were significantly worse in the Kishino-Hasegawa test as implemented in PAUP* [9] (α = 0.01).

**Figure 2:**
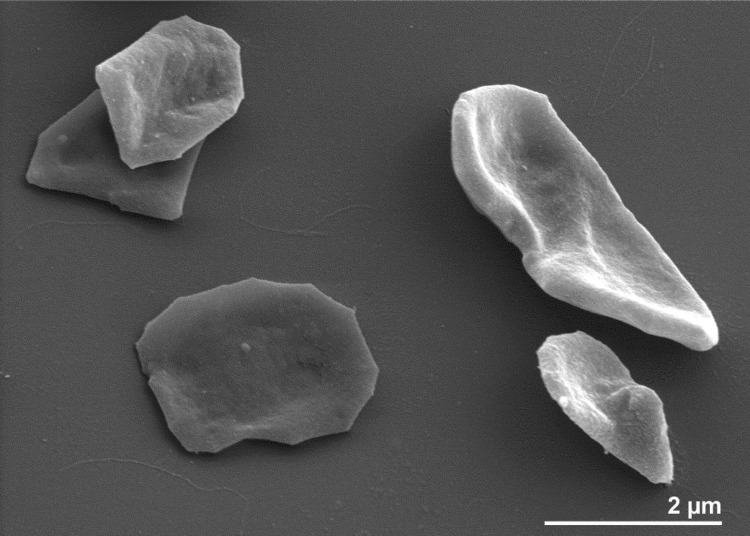
Scanning electron micrograph of *M. limicola* M3^T^

*M. limicola* M3^T^ cells stain Gram negative [1] and are plate-shaped with sharp crystal-like edges 1−3 µm long and 1−2 µm wide (Figure 2 and [1]). Weak motility was observed and motility genes were identified in the genome (see below). Polar tufts of flagella were also reported [1], but not visible in Figure 2. Granules with putative reserve material were observed in thin section EM images, as were curious ‘bone-shaped’ cells [1]. Cell envelopes consist of an S-layer glycoprotein with a hexagonal surface pattern [1]. Cultures grow with H_2_ or formate as sole substrates supplemented with ≥ 0.1% acetate essentially required [1]. Growth temperatures span from 17−41°C (optimum 40°C) in the presence of 0.4−5.4% NaCl (optimum 1%) [1]. A summary of the classification and features is presented in Table 1.

**Table 1:** Classification and general features of *M. limicola* M3^T^ according to the MIGS recommendations [16] published by the Genomic Standards Consortium [17].

**MIGS ID**	**Property**	**Term**	**Evidence code**
	Current classification	Domain *Bacteria*	TAS [18]
		Phylum *Euryarchaeota*	TAS [19]
		Class *Methanomicrobia*	TAS [20]
		Order *Methanomicrobiales*	TAS [21-25]
		Family *Methanomicrobiaceae*	TAS [21,22]
		Genus *Methanoplanus*	TAS [1,2]
		Species *Methanoplanus limicola*	TAS [1,2]
		Type strain M3	TAS [1]
	Gram stain	negative	TAS [1]
	Cell shape	plate-like	TAS [1]
	Motility	weakly motile	TAS [1]
	Sporulation	not reported	
	Temperature range	mesophile, 17-41°C	TAS [1]
	Optimum temperature	40°C	TAS [1]
	Salinity	0.4 - 5.4% NaCl (w/v), optimum 1.0%	TAS [5]
MIGS-22	Oxygen requirement	anaerobe	TAS [1]
	Carbon source	CO_2_, formate	TAS [1]
	Energy metabolism	methanogen, chemoorganotrophic	TAS [1]
MIGS-6	Habitat	swamps of fresh water and seawater	TAS [1]
MIGS-15	Biotic relationship	free living	TAS [1]
MIGS-14	Pathogenicity	none	NAS
	Biosafety level	1	TAS [26]
MIGS-23.1	Isolation	mud of drilling swamp	TAS [1]
MIGS-4	Geographic location	near Baia, Naples Area, Italy	TAS [1]
MIGS-5	Sample collection time	1981 or earlier	NAS
MIGS-4.1	Latitude	40.629	NAS
MIGS-4.2	Longitude	14.362	NAS
MIGS-4.3	Depth	not reported	
MIGS-4.4	Altitude	not reported	

Evidence codes - TAS: Traceable Author Statement (i.e., a direct report exists in the literature); NAS: Non-traceable Author Statement (i.e., not directly observed for the living, isolated sample, but based on a generally accepted property for the species, or anecdotal evidence). Evidence codes are from of the Gene Ontology project [27].

### Chemotaxonomy

No chemotaxonomical results were reported for strain M3^T^, except for an estimation of 47.5% for the G+C content of the genome determined by a melting point in 0.1 × SSC [1].

## Genome sequencing and annotation

### Genome project history

This organism was selected for sequencing on the basis of its phylogenetic position [28], and is part of the ***G****enomic* ***E****ncyclopedia of* ***B****acteria and* ***Archaea***** project [29]. The genome project is deposited in the Genomes On Line Database [10] and the complete genome sequence is deposited in GenBank. Sequencing, finishing and annotation were performed by the DOE Joint Genome Institute (JGI) using state of the art sequencing technology [30]. A summary of the project information is shown in Table 2.

**Table 2:** Genome sequencing project information

**MIGS ID**	**Property**	**Term**
MIGS-31	Finishing quality	Improved-high-quality-draft
MIGS-28	Libraries used	Three genomic libraries: one 454 pyrosequence standard library, one 454 PE library (5 kb insert size), one Illumina library
MIGS-29	Sequencing platforms	Illumina GAii, 454 GS FLX Titanium
MIGS-31.2	Sequencing coverage	834.5 × Illumina; 33.4 × pyrosequence
MIGS-30	Assemblers	Newbler version 2.3, Velvet 1.0.13, phrap version SPS - 4.24
MIGS-32	Gene calling method	Prodigal
	INSDC ID	CM001436, AHKP00000000
	GenBank Date of Release	January 24, 2012
	GOLD ID	Gi02923
	NCBI project ID	61291
	Database: IMG	2506381025
MIGS-13	Source material identifier	DSM 2279
	Project relevance	Tree of Life, GEBA

### Growth conditions and DNA isolation

*M. limicola* strain M3^T^, DSM 2279, was grown anaerobically under H_2_/CO_2_ gas phase in DSMZ medium 141 (*Methanogenium* medium; MMG medium + 0.1% acetate; substrate: H_2_ or formate; stimulated by YE or peptone, + vitamins) [31] at 35−40°C. DNA was isolated from 0.5-1 g of cell paste using MasterPure Gram-positive DNA purification kit (Epicentre MGP04100) following the standard protocol as recommended by the manufacturer with modification st/LALM for cell lysis as described in Wu *et al*. 2009 [29]. DNA is available through the DNA Bank Network [32].

### Genome sequencing and assembly

The genome was sequenced using a combination of Illumina and 454 sequencing platforms. All general aspects of library construction and sequencing can be found at the JGI website [33]. Pyrosequencing reads were assembled using the Newbler assembler (Roche). The initial Newbler assembly consisting of 760 contigs in ten scaffolds was converted into a phrap [34] assembly by making fake reads from the consensus, to collect the read pairs in the 454 paired end library. Illumina GAii sequencing data (3,470.2 Mb) was assembled with Velvet [35] and the consensus sequences were shredded into 1.5 kb overlapped fake reads and assembled together with the 454 data. The 454 draft assembly was based on 332.3 Mb 454 draft data and all of the 454 paired end data. Newbler parameters are -consed -a 50 -l 350 -g -m -ml 20. The Phred/Phrap/Consed software package [34] was used for sequence assembly and quality assessment in the subsequent finishing process. After the shotgun stage, reads were assembled with parallel phrap (High Performance Software, LLC). Possible mis-assemblies were corrected with gapResolution [33], Dupfinisher [36], or sequencing cloned bridging PCR fragments with subcloning. Gaps between contigs were closed by editing in Consed, by PCR and by Bubble PCR primer walks (J.-F. Chang, unpublished). A total of 159 additional reactions were necessary to close some gaps and to raise the quality of the final sequence. Illumina reads were also used to correct potential base errors and increase consensus quality using a software Polisher developed at JGI [37]. The error rate of the final genome sequence is less than 1 in 100,000. Together, the combination of the Illumina and 454 sequencing platforms provided 867.9 x coverage of the genome. The final assembly contained 421,665 pyrosequence and 44,481,858 Illumina reads.

### Genome annotation

Genes were identified using Prodigal [38] as part of the DOE-JGI [39] genome annotation pipeline, followed by a round of manual curation using the JGI GenePRIMP pipeline [40]. The predicted CDSs were translated and used to search the National Center for Biotechnology Information (NCBI) non-redundant database, UniProt, TIGRFam, Pfam, PRIAM, KEGG, COG, and InterPro databases. These data sources were combined to assert a product description for each predicted protein. Additional gene prediction analysis and functional annotation was performed within the Integrated Microbial Genomes - Expert Review (IMG-ER) platform [41].

## Genome properties

**Figure 3:**
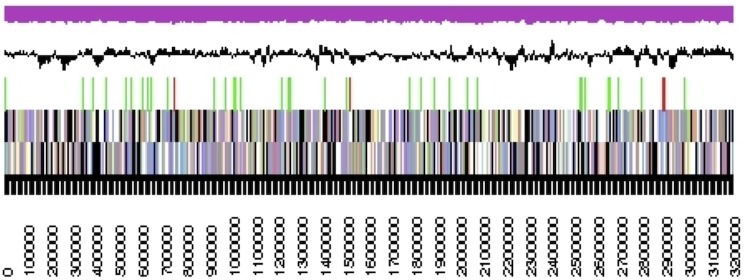
Graphical map of the chromosome. From bottom to the top: Genes on forward strand (colored by COG categories), Genes on reverse strand (colored by COG categories), RNA genes (tRNAs green, rRNAs red, other RNAs black), GC content (black), GC skew (purple/olive).

The genome consists of one scaffold (circularity not experimentally proven) of 3,200,946 bp length with a 42.2% G+C content (Table 3 and Figure 3). Of the 3,128 genes predicted, 3,064 were protein-coding genes, and 65 RNAs; 122 pseudogenes were also identified. The majority of the protein-coding genes (60.8%) were assigned a putative function while the remaining ones were annotated as hypothetical proteins. The distribution of genes into COGs functional categories is presented in Table 4.

**Table 3:** Genome Statistics

**Attribute**	**Value**	**% of Total**
Genome size (bp)	3,200,946	100.00%
DNA coding region (bp)	2,799,644	87.46%
DNA G+C content (bp)	1,350,606	42.20%
Number of replicons	1	
Extrachromosomal elements	0	
Total genes	3,129	100.00%
RNA genes	65	2.08%
rRNA operons	1*	
tRNA genes	56	1.79%
Protein-coding genes	3,064	97.92%
Pseudo genes	122	3.90%
Genes with function prediction (proteins)	1,901	60.75%
Genes in paralog clusters	1,568	50.11%
Genes assigned to COGs	2,204	70.44%
Genes assigned Pfam domains	2,149	68.68%
Genes with signal peptides	129	4.12%
Genes with transmembrane helices	748	23.91%
CRISPR repeats	0	

*but five genes for 5S rRNA

**Table 4:** Number of genes associated with the general COG functional categories

**Code**	**value**	**%age**	**Description**
J	155	6.5	Translation, ribosomal structure and biogenesis
A	1	0.0	RNA processing and modification
K	133	5.6	Transcription
L	129	5.4	Replication, recombination and repair
B	3	0.1	Chromatin structure and dynamics
D	17	0.7	Cell cycle control, cell division, chromosome partitioning
Y	0	0.0	Nuclear structure
V	33	1.4	Defense mechanisms
T	191	8.0	Signal transduction mechanisms
M	90	4.8	Cell wall/membrane biogenesis
N	79	3.3	Cell motility
Z	1	0.0	Cytoskeleton
W	0	0.0	Extracellular structures
U	27	1.1	Intracellular trafficking and secretion, and vesicular transport
O	82	3.4	Posttranslational modification, protein turnover, chaperones
C	173	7.2	Energy production and conversion
G	75	3.1	Carbohydrate transport and metabolism
E	147	6.1	Amino acid transport and metabolism
F	61	2.6	Nucleotide transport and metabolism
H	157	6.6	Coenzyme transport and metabolism
I	28	1.2	Lipid transport and metabolism
P	115	4.8	Inorganic ion transport and metabolism
Q	8	0.3	Secondary metabolites biosynthesis, transport and catabolism
R	355	14.8	General function prediction only
S	332	13.8	Function unknown
-	925	29.6	Not in COGs

## Insights into the genome sequence

### The G+C content in the genus *Methanoplanus*

When calculated from the genome sequences, the G+C content of *M. limicola* DSM 2279 amounts to 42.2%, whereas the previously published value, determined using traditional (“wet-lab”) techniques, is 47.5% [1]. Similarly, the G+C content of *M. petrolearius* was given as 50% [42], whereas the analysis of the genome sequence of the type strain SEBR 4837^T^ (DSM 11571) yielded 47.4% [11]. It was frequently stated in the literature that “organisms that differ by more than 10 mol% do not belong to the same genus and that 5 mol% is the common range found within a species” [43]. A recent study [44] has shown that when calculated from genome sequences the G+C content varies at most 1% within species and that larger variances are caused by the limitations of the traditional techniques for analyses. It has thus been recommended to conduct emendations of species descriptions in the case of discrepancies larger than 1%, and to also conduct emendations of genus descriptions if the species emendations yield values that do not fit into the range of the G+C content given in the literature for the respective genus [44].

### Considerations about the polyphyletic genus *Methanoplanus*

The phylogenetic tree presented in Figure 1 shows *Methanoplanus* as a polyphyletic taxon with the members of *Methanomicrobium* and *Methanolacinia* interspersed between the members of *Methanoplanus*. Given the high bootstrap support for the branches in that section of the phylogenetic tree, this situation calls for some attention, mainly due to the location of *M. petrolearius* [42]. The conflict between 16S rRNA gene data and the classification is significant, as revealed by the bootstrap values and the paired-site tests described above.

The problematic local structure of the phylogenetic tree might be caused by the fact that most of the five species located in the respective part of the tree were already decribed in the early days of Archaea research when only a limited number of reference sequences were available: *M. limicola* dates from 1982 [1], *M. endosymbiosus* from 1986 [45], *M. petrolearius* from 1997 [42], *M. paynteri from* 1983 [46] (renamed in 1989 [47]), and *Methanomicrobium mobilis* even from 1968 [48]. State-of-the-art techniques for the initial taxonomic characterization of the then novel bacteria were much less advanced than today, e.g. Sanger sequencing had just been invented (in 1977) when *M. limicola* was characterized with DNA-RNA hybridizations as decisive technique [49], and still not yet generally used for taxonomic work when *M. endosymbiosus* was characterized four years later. When the latest of the three *Methanoplanus* species with a validly published name, *M. petrolearius*, was added in 1997 16S rRNA sequences were used, but the ones from *M. paynteri* (closest neighbor in the phylogenetic tree in Figure 1) and *M. mobilis* were not yet available or at least not used for comparative analyses [42]. 

The completion of the Sequencing Orphan Species (SOS) initiative early last year [50], closed the last gaps in the availability of high-quality 16S rRNA reference sequences for phylogenetic trees. However, a decade after the first genome-based investigations into the history of the domain *Archaea* [51] and the systematic overview of their evolution, physiology, and molecular biology [52], a significant fraction of draft genome sequences as such generated in the genomic Encyclopedia of *Bacteria* and *Archaea* [29] are still very much needed to cover all of the diversity of the *Archaea*, especially from difficult-to-grow organisms and from type strains of remote clades such as the *Methanomicrobiaceae*.

With all these limitations, a closer inspection of the positions of the members of *Methanoplanus* in Figure 1 might still be worthwhile. *M. petrolearius* appears to be clearly separated from the other two members of the genus, *M. limicola* and *M. endosymbiosus*, but closely linked to *M. paynteri with* a 99.8% 16S rRNA gene sequence identity. Table 5 shows a summary of the features of all members of the genera *Methanoplanus* and *Methanolacinia,* indicating that based on the higher optimal growth temperature, the lack of observed flagella and observed motility (although the flagellin genes are encoded in the genome), the usage of CO_2_+2-propanol as a substrate, and the higher G+C content of the genome [42], *M. petrolearius* clusters rather with *M. paynteri than* with the other two members of *Methanoplanus.*

**Table 5:** Features of the type strains within the genera *Methanoplanus* and *Methanolacinia*.

	*M. limicola* [1] DSM 2279	*M. endosymbiosus* [45] DSM 3599	*M. petrolearius* [42] DSM 11571	*M. paynteri* [46,47] DSM 2545
Source	swamp	marine ciliate	oil well	marine sediment
Temperature range (T_opt_) °C	17−41 (32)	16−36 (32)	28−43 (37)	unknown (40)
motility	motile, flagella	flagella reported	non-motile	non-motile
pH range (pH_opt_)	ND (6.5−7.5)	6.1−8.0 (6.8−7.3)	5.3−8.2 (7.0)	ND
NaCl conc. % (opt.)	0.4−5.4 (1)	0−4.5 (1.5)	0−5 (1−3)	
Substrates used	H_2_+CO_2_, formate	H_2_+CO_2_, formate	H_2_+CO_2_, formate, CO_2_+2-propanol	H_2_+CO_2_, CO_2_+2-propanol, CO_2_+2-butanol, CO_2_+2-cyclopentanol
G+C content	42.2% genome (was 47.5% pre-genome)	38.7% melting curve	47.4% genome (was 50% pre-genome)	44.8% buoyant density

Although the genome sequence of *M. petrolearius* SEBR 4847^T^ (DSM 11571) was recently published [11], the one for *Ml. paynteri* was still lacking, as well as information about a wet lab DNA-DNA hybridization (DDH) between the type strains of the two species. Given the high degree of 16S rRNA sequence identity between the two strains (99.8%), established thresholds of species delimitations, 97% [53], even under recently published relaxed recommendations, 98.2−99% [54], definitely demands such an analysis for the purpose of species discrimination. Whereas the rather large difference of 2.6% in the G+C content of the two genomes (Table 5, based on currently available mixed data from genome sequence and buoyant density measurement) predicts a rather low DDH value as the outcome of such an experiment, the recently observed significant deviations between previously published G+C values and G+C values inferred from genome sequences [44] do not, however, allow for definitive conclusions from the difference in G+C values.

For this reason, we have obtained a draft genome sequence for *M. paynteri* DSM 2546^T^ using Illumina-MiSeq as a sequencing platform in order to obtain paired-end reads of 250 bp and Velvet [35] for the assembly. The draft genome comprised 54 contigs and is available from NCBI under the accession number AXDV00000000 and from IMG under the object ID *pending*. Digital DDH similarities between *Ml. paynteri* DSM 2546^T^ (AXDV00000000) and *M. petrolearius* SEBR 4847^T^ (DSM 11571, CP002117) were calculated with the GGDC web server [55,56] version 2.0 [57] under the recommended settings. The inter-genomic distance (formula 2) was 0.0753, corresponding to a DDH estimate of 48.50% ± 2.61%. The probability of a DDH value > 70% was accordingly only 0.1514.

In conclusion, from the topology of 16S rRNA gene sequence-based phylogenetic tree supported by the distribution of the characteristic features listed in Table 4 we can conclude that strain SEBR 4847^T^ should rather be classified as a member of the genus *Methanolacinia* than as *M. petrolearius*, whereas the digital DDH results clearly indicate that *Ml. paynteri* (represented by the type strain G-2000, DSM 2545) and *M. petrolearius* (represented by the type strain SEBR 4847, DSM 11571) are distinct species. Thus, we propose *Methanolacinia petrolearia* comb. nov. to accommodate *M. petrolearius*, with SEBR 4847 being the type strain.

The situation between *M. limicola* (type species of *Methanoplanus*) and *M. endosymbiosus* is only slightly better than the relationship between *M. paynteri* and *M. petrolearius* discussed above. Based on the above reported Greengenes analysis the 16S rRNA gene sequences of the two type strains show 99.5% sequence identity and an HSP coverage of 99.7%. Again, by all accepted standards of species discrimination [53,54] such a close similarity would call for a DDH experiment to resolve the close relationship, but such data are not available. Also a digital DDH cannot be performed because only the genome sequence of *M. limicola* presented here is available, but not that of DSM 3599, the type strain of *M. endosymbiosus.* Table 5 indicates that the two strains share almost all of the listed features (except habitat), except for a 3.5% difference in the G+C content, which, in case it would be confirmed and not biased by a technical artifact in the melting curve measurement done for *M. endosymbiosus*, indicated a sufficiently low level of DDH to distinguish the two species [44]. Nevertheless, the probability that the digital DDH value between the two type strains might surpass the 70% species discrimination (once the genome sequence of *M. endosymbiosus* is resolved) threshold cannot be neglected. It might be too early to draft the obituary for *M. endosymbiosus*, but it is better to be prepared in case the once trispecific polyphyletic genus *Methanoplanus* becomes monospecific, an event that may occur once the drafts of all needed type strain genomes (the core objective of GEBA) are deciphered. Depending on the availability of enough cell material, *M. endosymbiosus* should now be scheduled as a sequencing target for the upcoming phases the GEBA, *e.g*. the Genomic Encyclopedia of Type Strains, Phase I: the one thousand microbial genomes (KMG-I) projects [58], to resolve the question about the exact relationship between *M. limicola* and *M. endosymbiosus*.

## Taxonomic consequences

As explained in detail above, the differences in the reported G+C contents of *M. limicola* and *M. petrolearius* to the ones calculated from their genome sequences justifies an emendation of the species descriptions. Moreover, *M. petrolearius* should be placed within the genus *Methanolacinia*. The descriptions of the two genera should be emended accordingly.

### Emended description of the species *Methanoplanus limicola* Wildgruber *et al.* 1982

The description of the species *Methanoplanus limicola* is the one given by Wildgruber *et al.* 1982 [1], with the following modification.

The G+C content is 42%.

### Emended description of the species *Methanoplanus petrolearius* Ollivier *et al.* 1997

The description of the species *Methanoplanus petrolearius* is the one given by Ollivier *et al.* 1997 [42], with the following modification.

The G+C content is 47%.

## Description of *Methanolacinia petrolearia*, comb. nov.

Basonym: *Methanoplanus petrolearius* Ollivier *et al*. 1997

The description of the species is the same as given for *Methanoplanus petrolearius* Ollivier et al. 1997 with the emendation given above.

### Emended description of the genus *Methanoplanus*

The description is the one given by Wildgruber *et al*. [1] with the following modifications:

The G+C content is 39-42%.

### Emended description of the genus *Methanolacinia*

The description is the one given by Zellner *et al*. [47] with the following modifications:

The G+C content is 45-47%.
